# Multivariate systems of nonexpansive operator equations and iterative algorithms for solving them in uniformly convex and uniformly smooth Banach spaces with applications

**DOI:** 10.1186/s13660-018-1629-7

**Published:** 2018-02-13

**Authors:** Yongchun Xu, Jinyu Guan, Yanxia Tang, Yongfu Su

**Affiliations:** 1 Department of Mathematics, Hebei North University, Zhangjiakou, China; 2 Department of Mathematics, Tianjin Polytechnic University, Tianjin, China

**Keywords:** Uniformly convex, Uniformly smooth, Banach space, Systems of nonexpansive operator equations, Solution, Iterative algorithms

## Abstract

We prove some existence theorems for solutions of a certain system of multivariate nonexpansive operator equations and calculate the solutions by using the generalized Mann and Halpern iterative algorithms in uniformly convex and uniformly smooth Banach spaces. The results of this paper improve and extend the previously known ones in the literature.

## Introduction and preliminaries

Multivariate mathematical analysis is an important branch in mathematical fields and applied science fields. A system of nonlinear operator equations is an essential tool in the broader fields of science and technology. It is also an important method in pure and applied mathematics. Many structures of mathematics can be expressed in the form of fixed point equations. For example, equilibrium problems, variational inequalities, convex optimization, split feasibility problems, and inclusion problems are equivalent to relatively fixed point problems. Furthermore, generalized equilibrium problems, generalized variational inequalities, generalized convex optimization, generalized split feasibility problems, and generalized inclusion problems are equivalent to relatively fixed point equation or systems of nonlinear operator equations (see [1–9]).

Recently, multivariate fixed point theorems of *N*-variable nonlinear mappings have been studied by some authors. Many interesting results and their applications have been also given. In 2014, Lee and Kim [5] proved multivariate coupled fixed point theorems on ordered partial metric spaces. In 2016, Su, Petruşel, and Yao [7] presented the concept of a multivariate fixed point and proved a multivariate fixed point theorem for *N*-variable contraction mappings, which further generalizes the Banach contraction mapping principle. In 2016, Luo, Su, and Gao [6] presented the concept of a multivariate best proximity point and proved multivariate best proximity point theorems in metric spaces for *N*-variable contraction mappings. In 2017, Xu et al. [8] presented the concept of a multivariate contraction mapping in a locally convex topological vector space and proved the multivariate contraction mapping principle in such spaces. In 2017, Guan et al. [4] studied a certain system of *N*-fixed point operator equations with *N*-pseudo-contractive mapping in reflexive Banach spaces and proved existence theorems of solutions. In 2017, Tang et al. [9] studied a certain system of *N*-variable variational inequalities and proved existence theorems of solutions.

Our purpose in this paper is to prove some existence theorems for solutions of a certain system of the multivariate nonexpansive operator equations and to calculate the solutions by using the generalized Mann and Halpern iterative algorithms in uniformly convex and uniformly smooth Banach spaces. The results of this paper improve and extend the previously known ones in the literature.

The following classical theorems are useful for the results of this paper.

### Theorem 1.1

(Browder and Göhde fixed point theorem [10])

*Let* *X* *be a real uniformly convex Banach space*, *and let* *C* *be a nonempty closed convex bounded subset of* *X*. *Then every nonexpansive mapping* $T: C\rightarrow C$ *has a fixed point*.

Mann’s iterative process was initially introduced in 1953 by Mann [11]. Mann’s iterative scheme is an important iterative scheme to study the class of nonexpansive mappings. The following is a representative result in recent years.

### Theorem 1.2

([12, 13])

*Let* *C* *be a nonempty closed convex subset of a real uniformly convex Banach space* *X*, *and let* $T: C\rightarrow C$ *be a nonexpansive mapping with nonempty fixed point set* $F(T)$. *Let* $\{\alpha_{n}\} \subset[0,1]$ *be a sequence of numbers such that* $0< a\leq\alpha_{n}\leq b<1$. *Then*, *for any given* $x_{0} \in X$, *the iterative processes* $\{x_{n}\}$ *defined by*

$$x_{n+1}=\alpha_{n} x_{n}+(1-\alpha_{n})Tx_{n} $$

*converges weakly to a fixed point of* *T*.

Halpern’s iterative scheme is also an important iterative scheme to study the class of nonexpansive mappings. Halpern’s iterative process was initially introduced in 1967 by Halpern in the framework of Hilbert spaces [14]. For any $u, x_{0} \in C$, the sequence $\{x_{n}\}$ is defined by

H $$ x_{n+1}=\alpha_{n} u+(1-\alpha_{n})Tx_{n}, \quad u \in C, $$

where $\{\alpha_{n}\} \subset[0,1]$. He proved that the sequence $\{x_{n}\} $ converges weakly to a fixed point of *T* when $\alpha_{n}=n^{-\alpha}$, $\alpha\in(0,1)$. In 1997, Lions [15] further proved that the sequence $\{x_{n}\}$ converges strongly to a fixed point of *T* in a Hilbert space if $\{\alpha_{n}\}$ satisfies the following conditions:

(C_1_): $\sum_{n=1}^{\infty}\alpha_{n}=\infty$;
(C_2_): $\lim_{n\rightarrow\infty}\alpha_{n}=0$;
(C_3_): $\lim_{n\rightarrow\infty}\frac{|\alpha_{n+1}-\alpha _{n}|}{\alpha^{2}_{n+1}}=0$.

However, in [11], the real sequence $\{\alpha_{n}\}$ excluded the canonical choice $\alpha_{n}=\frac{1}{n+1}$. In 1992, Wittmann [16] proved, still in Hilbert spaces, the strong convergence of (H) to a fixed point of *T* if $\{\alpha_{n}\}$ satisfies the following conditions:

(C_1_): $\sum_{n=1}^{\infty}\alpha_{n}=\infty$;
(C_2_): $\lim_{n\rightarrow\infty}\alpha_{n}=0$;
(C_4_): $\lim_{n\rightarrow\infty}\sum_{n=1}^{\infty}|\alpha _{n+1}-\alpha_{n}|<+ \infty$.

The strong convergence of Halpern’s iteration to a fixed point of *T* has also been proved in Banach spaces [17–19]. In 1997, Shioji and Takahashi [18] extended Wittmann’s result to Banach spaces. In 2002, Xu [19] obtained a strong convergence theorem where $\{\alpha_{n}\}$ satisfies conditions (C_1_), (C_2_), and the following condition:

(C_5_): $\lim_{n\rightarrow\infty}\frac{\alpha_{n+1}-\alpha_{n}}{\alpha_{n}}=0$.

### Theorem 1.3

([19])

*Let* *C* *be a nonempty closed convex subset of a real uniformly smooth Banach space* *X*, *and let* $T: C\rightarrow C$ *be a nonexpansive mapping with nonempty fixed point set* $F(T)$. *Let* $\{\alpha_{n}\} \subset[0,1]$ *be a sequence of numbers satisfying the following conditions*:

(C_1_): $\sum_{n=1}^{\infty}\alpha_{n}=\infty$;
(C_2_): $\lim_{n\rightarrow\infty}\alpha_{n}=0$;
(C_5_): $\lim_{n\rightarrow\infty}\frac{\alpha_{n+1}-\alpha_{n}}{\alpha_{n}}=0$.

*Then*, *for any given* $x_{0} \in C$, *the iterative processes* $\{x_{n}\}$ *defined by*

$$x_{n+1}=\alpha_{n} u+(1-\alpha_{n})Tx_{n}, \quad u \in C $$

*converges strongly to a fixed point of* *T*.

## Cartesian product of uniformly convex Banach spaces

### Definition 2.1

A Banach space $(X,\|\cdot\|)$ is said to be uniformly convex if

$$x_{n}, y_{n} \in X,\qquad \|x_{n}\|\leq1,\qquad \|y_{n}\|\leq1,\quad \mbox{and}\quad \lim_{n\rightarrow\infty} \|x_{n}+y_{n}\|=2 $$

imply

$$\lim_{n\rightarrow\infty}\|x_{n}-y_{n}\|=0. $$

### Theorem 2.2

*Let* *X* *be a Banach space with norm* $\|\cdot\| $, *and let* $X^{N}=X\times X\times \cdots\times X$ *be the Cartesian product space of* *X*. *Let*

$$\|x \|_{*}=\sqrt{ \sum_{i=1}^{N}\| x_{i}\|^{2}},\quad x=(x_{1},x_{2}, \ldots,x_{N}) \in X^{N}. $$

*Then* $(X^{N}, \|\cdot\|_{*})$ *is a Banach space*. *If* $(X^{N}, \|\cdot\|_{*})$ *is uniformly convex*, *then* *X* *must be uniformly convex*.

### Proof

We only need to prove the uniformly convexity of $(X, \|\cdot\|)$. Let

$$x_{n}, y_{n} \in X,\qquad \|x_{n}\|\leq1,\qquad \|y_{n}\|\leq1,\quad \mbox{and}\quad \lim_{n\rightarrow\infty} \|x_{n}+y_{n}\|=2 $$

for all $n\geq1$. Then

$$\begin{aligned}& \bigl\Vert (x_{n}, 0,0, \ldots,0) \bigr\Vert _{*}= \Vert x_{n} \Vert \leq1, \\& \bigl\Vert (y_{n}, 0,0, \ldots,0) \bigr\Vert _{*}= \Vert y_{n} \Vert \leq1, \end{aligned}$$

and

$$\begin{aligned}& \lim_{n\rightarrow\infty} \bigl\Vert (x_{n}, 0,0, \ldots,0)+(y_{n}, 0,0, \ldots ,0) \bigr\Vert _{*} \\& \quad =\lim_{n\rightarrow\infty} \bigl\Vert (x_{n}+y_{n}, 0,0, \ldots,0) \bigr\Vert _{*} \\& \quad = \lim_{n\rightarrow\infty} \Vert x_{n}+y_{n} \Vert =2. \end{aligned}$$

Since $(X^{N}, \|\cdot\|_{*})$ is uniformly convex, we have

$$\begin{aligned}& \lim_{n\rightarrow\infty} \Vert x_{n}-y_{n} \Vert \\& \quad = \lim_{n\rightarrow\infty} \bigl\Vert (x_{n}, 0,0, \ldots,0)-(y_{n}, 0,0, \ldots ,0) \bigr\Vert _{*} \\& \quad =\lim_{n\rightarrow\infty} \bigl\Vert (x_{n}-y_{n}, 0,0, \ldots,0) \bigr\Vert _{*}=0. \end{aligned}$$

Hence *X* is uniformly convex. This completes the proof. □

From Theorem 2.2 we get the following corollary.

### Corollary 2.3

*Let* *X* *be a nonuniformly convex Banach space with norm* $\|\cdot\|$. *Let* $X^{N}=X\times X\times \cdots\times X$ *be the Cartesian product space of* *X*. *Let*

$$\|x \|_{*}=\sqrt{ \sum_{i=1}^{N}\| x_{i}\|^{2}}, \quad x=(x_{1},x_{2}, \ldots ,x_{N}) \in X^{N}. $$

*Then* $(X^{N}, \|\cdot\|_{*})$ *is a nonuniformly convex Banach space*.

### Theorem 2.4

*Let* *X* *be a Banach space with norm* $\|\cdot\| $, *and let* $X^{N}=X\times X\times \cdots\times X$ *be the Cartesian product space of* *X*. *Let*

$$\|x \|_{*}= \sum_{i=1}^{N}\| x_{i} \|,\quad x=(x_{1},x_{2},\ldots,x_{N}) \in X^{N}. $$

*Then* $(X^{N}, \|\cdot\|_{*})$ *is a nonuniformly convex Banach space*.

### Proof

We only need to check that $(X^{N}, \|\cdot\|_{*})$ is not uniformly convex. Let $u \in X$ be such that $\|u\|=1$ and

$$x_{n}=(u, 0,0, \ldots,0), y_{n}=(0, u, 0,0, \ldots,0) \in X^{N} $$

for all $n=1,2,\ldots$ . Then

$$\|x_{n}\|_{*}= 1,\qquad \|y_{n}\|_{*}= 1, \qquad \|x_{n}+y_{n}\|_{*}=2 $$

for all $n\geq1$. However, $\|x_{n}-y_{n}\|_{*}=2$ for all $n=1,2,\ldots$ . Hence $(X^{N}, \|\cdot\|_{*})$ is not uniformly convex. This completes the proof. □

### Theorem 2.5

*Let* *X* *be a Banach space with norm* $\|\cdot\| $, *and let* $X^{N}=X\times X\times \cdots\times X$ *be the Cartesian product space of* *X*. *Let*

$$\|x \|_{*}=\max_{1\leq i \leq N}\| x_{i}\|, \quad x=(x_{1},x_{2},\ldots,x_{N}) \in X^{N}. $$

*Then* $(X^{N}, \|\cdot\|_{*})$ *is a nonuniformly convex Banach space*.

### Proof

We only need to check that $(X^{N}, \|\cdot\|_{*})$ is not uniformly convex. Let $u \in X$ be such that $\|u\|=1$ and

$$x_{n}=(u, 0,0, \ldots,0), y_{n}=(u, u, 0,0, \ldots,0) \in X^{N} $$

for all $n=1,2,\ldots$ . Then

$$\|x_{n}\|_{*}= 1,\qquad \|y_{n}\|_{*}= 1,\qquad \|x_{n}+y_{n}\|_{*}=2 $$

for all $n\geq1$. However, $\|x_{n}-y_{n}\|_{*}=1$ for all $n=1,2,\ldots$ . Hence $(X^{N}, \|\cdot\|_{*})$ is not uniformly convex. This completes the proof. □

### Open question 2.6

Let *X* be a Banach space with norm $\|\cdot\| $, and let $X^{N}=X\times X\times \cdots\times X$ be the Cartesian product space of *X*. Let

$$\|x \|_{*}=\sqrt{ \sum_{i=1}^{N}\| x_{i}\|^{2}}, \quad x=(x_{1},x_{2}, \ldots ,x_{N}) \in X^{N}. $$

Under which conditions $(X^{N}, \|\cdot\|_{*})$ is a uniformly convex Banach space?

## Cartesian product of uniformly smooth Banach spaces

### Definition 3.1

A Banach space $(X,\|\cdot\|)$ is said to be uniformly smooth if for any real number $\varepsilon>0$, there exists a real number $\delta>0$ such that

$$\|x\|=1,\qquad 0< \|y\|< \delta,\quad x \in X, y \in X, $$

implies

$$\frac{\|x+y\|+\|x-y\|-2}{\|y\|}< \varepsilon. $$

### Theorem 3.2

*Let* *X* *be a Banach space with norm* $\|\cdot\| $, *and let* $X^{N}=X\times X\times \cdots\times X$ *be the Cartesian product space of* *X*. *Let*

$$\|x \|_{*}=\sqrt{ \sum_{i=1}^{N}\| x_{i}\|^{2}},\quad x=(x_{1},x_{2}, \ldots ,x_{N}) \in X^{N}. $$

*Then* $(X^{N}, \|\cdot\|_{*})$ *is a Banach space*. *If* $(X^{N}, \|\cdot\|_{*})$ *is uniformly smooth*, *then* *X* *must be uniformly smooth*.

### Proof

We only need to prove the uniform smoothness of $(X, \|\cdot\|)$. Since $(X^{N}, \|\cdot\|_{*})$ is uniformly smooth, then for any real number $\varepsilon>0$, there exists a real number $\delta>0$ such that

$$\Vert x \Vert = \bigl\Vert (x,0,\ldots,0) \bigr\Vert _{*}=1,\qquad 0< \Vert y \Vert = \bigl\Vert (y,0,\ldots,0) \bigr\Vert _{*}< \delta,\quad x \in X, y \in X, $$

implies

$$\frac{\|x+y\|+\|x-y\|-2}{\|y\|}=\frac{\|(x+y,0,\ldots,0)\|_{*}+\|(x-y, 0,\ldots,0)\|_{*}-2}{\|(y,0)\|_{*}}< \varepsilon. $$

Hence *X* is uniformly smooth. This completes the proof. □

From Theorem 3.2 we get the following corollary.

### Corollary 3.3

*Let* *X* *be a nonuniformly smooth Banach space with norm* $\|\cdot\|$, *and let* $X^{N}=X\times X\times \cdots\times X$ *be the Cartesian product space of* *X*. *Let*

$$\|x \|_{*}=\sqrt{ \sum_{i=1}^{N}\| x_{i}\|^{2}}, \quad x=(x_{1},x_{2}, \ldots ,x_{N}) \in X^{N}. $$

*Then* $(X^{N}, \|\cdot\|_{*})$ *is a nonuniformly smooth Banach space*.

### Theorem 3.4

*Let* *X* *be a Banach space with norm* $\|\cdot\| $, *and let* $X^{N}=X\times X\times \cdots\times X$ *be the Cartesian product space of* *X*. *Let*

$$\|x \|_{*}= \sum_{i=1}^{N}\| x_{i} \|,\quad x=(x_{1},x_{2},\ldots,x_{N}) \in X^{N}. $$

*Then* $(X^{N}, \|\cdot\|_{*})$ *is a nonuniformly smooth Banach space*.

### Proof

We only need to check that $(X^{N}, \|\cdot\|_{*})$ is not uniformly smooth. Let $u \in X$ be such that $\|u\|=1$ and

$$x=(u, 0,0, \ldots,0), y_{n}=\biggl(0, \frac{1}{n}u, 0,0, \ldots,0\biggr) \in X^{N} $$

for all $n=1,2,\ldots $ . In this case,

$$\frac{\|x+y\|_{*}+\|x-y\|_{*}-2}{\|y\|_{*}}=\frac{2\frac{n+1}{n}-2}{\frac{1}{n}}=2 $$

for all $n=1,2,\ldots$ . Hence $(X^{N}, \|\cdot\|_{*})$ is not uniformly smooth. This completes the proof. □

### Theorem 3.5

*Let* *X* *be a Banach space with norm* $\|\cdot\| $, *and let* $X^{N}=X\times X\times \cdots\times X$ *be the Cartesian product space of* *X*. *Let*

$$\|x \|_{*}=\max_{1\leq i \leq N}\| x_{i}\|,\quad x=(x_{1},x_{2},\ldots,x_{N}) \in X^{N}. $$

*Then* $(X^{N}, \|\cdot\|_{*})$ *is a nonuniformly smooth Banach space*.

### Proof

We only need to check that, $(X^{N}, \|\cdot\|_{*})$ is not uniformly smooth. Let $u \in X$ be such that $\|u\|=1$ and

$$x=(u, u,0, \ldots,0), y_{n}=\biggl(\frac{1}{n}u,, \frac{-1}{n}u, 0,0, \ldots,0\biggr) \in X^{N} $$

for all $n=1,2,\ldots $ . In this case,

$$\frac{\|x+y\|_{*}+\|x-y\|_{*}-2}{\|y\|_{*}}=\frac{2\frac{n+1}{n}-2}{\frac{1}{n}}=2 $$

for all $n=1,2,\ldots$ . Hence $(X^{N}, \|\cdot\|_{*})$ is not uniformly smooth. This completes the proof. □

### Open question 3.6

Let *X* be a Banach space with norm $\|\cdot\| $, and let $X^{N}=X\times X\times \cdots\times X$ be the Cartesian product space of *X*. Let

$$\|x \|_{*}=\sqrt{ \sum_{i=1}^{N}\| x_{i}\|^{2}},\quad x=(x_{1},x_{2}, \ldots ,x_{N}) \in X^{N}. $$

Under which conditions $(X^{N}, \|\cdot\|_{*})$ is a uniformly smooth Banach space?

## Results and discussion

### Definition 4.1

Let *X* be a real normed space, and let *C* a nonempty subset of *X*. Let $T: C^{N}\rightarrow C$ be an *N*-variable mapping satisfying the following condition:

$$\|Tx-Ty\|\leq\sqrt{ \frac{1}{N}\sum _{j=1}^{N} \|x_{j}-y_{j} \|^{2}}, $$

where $x=(x_{1},x_{2},\ldots,x_{N}), y=(y_{1},y_{2},\ldots,y_{N}) \in C^{N}$. Then *T* is said to be nonexpansive.

### Theorem 4.2

*Let* $(X, \|\cdot\|)$ *be a real uniformly convex Banach space*, *and let* *C* *be a nonempty closed convex bounded subset of* *X*. *Let* $T_{i}: C^{N}\rightarrow C$ *be an* *N*-*variable nonexpansive mapping for all* $i=1,2,\ldots,N$. *Let*

$$\|x\|_{*}= \sqrt{ \sum_{i=1}^{N} \|x_{i}\|^{2}} $$

*for all* $x=(x_{1},x_{2},\ldots,x_{N}) \in X^{N}$. *Assume that* $(X^{N}, \|\cdot\|_{*})$ *is a uniformly convex Banach space*. *Then there exists an element* $p=(x_{1},x_{2}, \ldots, x_{N})\in C^{N}$ *such that*

4.1 $$ \textstyle\begin{cases} T_{1}(x_{1},x_{2},\ldots,x_{N})=x_{1}, \\ T_{2}(x_{1},x_{2},\ldots,x_{N})=x_{2}, \\ \ldots, \\ T_{i}(x_{1},x_{2},\ldots,x_{N})=x_{i}, \\ \ldots, \\ T_{N}(x_{1},x_{2},\ldots,x_{N})=x_{N}. \end{cases} $$

### Proof

The operator $T^{*}: C^{N}\rightarrow C^{N}$ is defined by

$$T^{*} :(x_{1},x_{2},\ldots,x_{N}) \mapsto(z_{1}, z_{2},\ldots ,z_{N}), $$

for all $x=(x_{1},x_{2},\ldots,x_{N}) \in C^{N}$, where

$$\begin{aligned}& z_{1}=T_{1}(x_{1},x_{2}, \ldots,x_{N}), \\& z_{2}=T_{2}(x_{1},x_{2}, \ldots,x_{N}), \\& \ldots, \\& z_{N}=T_{N}(x_{1},x_{2}, \ldots,x_{N}). \end{aligned}$$

Then, for all $x=(x_{1},x_{2},\ldots,x_{N}), y=(y_{1},y_{2},\ldots,y_{N}) \in C^{N}$, we have that

$$\begin{aligned} \bigl\| T^{*}x-T^{*}y\bigr\| _{*}&=\sqrt{ \sum_{i=1}^{N} \|T_{i}x-T_{i}y\|^{2}} \\ & \leq\sqrt{ \sum_{i=1}^{N} \frac{1}{N}\sum_{j=1}^{N} \|x_{j}-y_{j}\|^{2}} \\ & \leq\sqrt{ \sum_{j=1}^{N} \|x_{j}-y_{j}\|^{2}} \\ & = \|x-y\|_{*}. \end{aligned}$$

Hence $T^{*}$ is a nonexpansive mapping from the nonempty closed convex bounded subset $C^{N}$ into itself in the uniformly convex Banach space $(X^{N}, \|\cdot\|_{*})$. By Theorem 1.1 we claim that $T^{*}$ has a fixed point

$$p=(p_{1},p_{2},\ldots,p_{N}) \in C^{N}, $$

that is,

$$T^{*} (p_{1},p_{2},p_{3},\ldots,p_{N}) = (p_{1}, p_{2}, p_{3},\ldots,p_{N}). $$

From the definition of $T^{*}$ we have

$$\begin{aligned}& p_{1}=T_{1}(p_{1},p_{2}, \ldots,p_{N}), \\& p_{2}=T_{2}(p_{1},p_{2}, \ldots,p_{N}), \\& \ldots, \\& p_{N}=T_{N}(p_{1},p_{2}, \ldots,p_{N}). \end{aligned}$$

This completes the proof. □

### Lemma 4.3

([14])

$(X^{N},\|\cdot\|_{*})^{*}=((X,\|\cdot\|)^{*})^{N}$.

### Lemma 4.4

*Let* $(X,\|\cdot\|)$ *be a Banach space that satisfies Opial’s condition*. *Let* $X^{N}=X\times X\times \cdots\times X$ *be the Cartesian product space of* *X*. *Let*

$$\|x \|_{*}= \sqrt{\sum_{i=1}^{N}\| x_{i}\|^{2} }, \quad x=(x_{1},x_{2}, \ldots ,x_{N}) \in X^{N}. $$

*Then* $(X^{N}, \|\cdot\|_{*})$ *satisfies Opial’s condition*.

### Proof

Let

$$x_{n}=(x_{1,n},x_{2,n},\ldots,x_{N,n}) , \quad n=1,2,3, \ldots, $$

be a sequence converging weakly to a point

$$x=(x_{1},x_{2},\ldots,x_{N}) $$

in Banach space $(X^{N}, \|\cdot\|_{*})$. From Lemma 4.3 we know that $\{x_{i,n}\}$ converges weakly to $x_{i}$ for all $i=1,2, \ldots, N$. Since $(X,\|\cdot\|)$ satisfies Opial’s condition, we have that

$$\limsup_{n\rightarrow\infty}\|x_{i,n}-x_{i}\|< \limsup _{n\rightarrow\infty }\|x_{i,n}-y_{i}\| $$

for any

$$y=(y_{1},y_{2},\ldots,y_{N}) \in \bigl(X^{N}, \|\cdot\|_{*}\bigr) $$

not equal to *x*, which implies that

$$\begin{aligned} \limsup_{n\rightarrow\infty}\|x_{n}-x\|_{*}&=\limsup _{n\rightarrow\infty }\sqrt{\sum_{i=1}^{N} \|x_{i,n}-x_{i}\|} \\ &< \limsup_{n\rightarrow\infty}\sqrt{\sum _{i=1}^{N}\|x_{i,n}-y_{i}\|} \\ &=\limsup_{n\rightarrow\infty}\|x_{n}-y\|_{*} . \end{aligned}$$

Then $(X^{N}, \|\cdot\|_{*})$ satisfies Opial’s condition. This completes the proof. □

### Theorem 4.5

*Let* *X* *be a real uniformly convex Banach space that satisfies Opial’s condition*, *and let* *C* *a nonempty closed convex bounded subset of* *X*. *Let* $T_{i}: C^{N}\rightarrow C$ *be an* *N*-*variable nonexpansive mapping for all* $i=1,2,\ldots,N$. *Let*

$$\|x\|_{*}= \sqrt{ \sum_{i=1}^{N} \|x_{i}\|^{2}} $$

*for all* $x=(x_{1},x_{2},\ldots,x_{N}) \in X^{N}$. *Assume that* $(X^{N}, \|\cdot\|_{*})$ *is a uniformly convex Banach space*. *Then*

1. *there exists an element* $p=(p_{1},p_{2}, \ldots, p_{N})\in C^{N}$ *such that*
  $$\textstyle\begin{cases} T_{1}(p_{1},p_{2},\ldots,p_{N})=p_{1}, \\ T_{2}(p_{1},p_{2},\ldots,p_{N})=p_{2}, \\ \ldots, \\ T_{i}(p_{1},p_{2},\ldots,p_{N})=p_{i}, \\ \ldots, \\ T_{N}(p_{1},p_{2},\ldots,p_{N})=p_{N}; \end{cases} $$
2. *for any* $x_{0}=(x_{1,0},x_{2,0},x_{3,0},\ldots,x_{N,0}) \in C^{N}$, *the iterative sequence* $\{x_{i,n}\}\subset X$ *defined by*
  $$x_{i, n+1}=\alpha_{n} x_{i,n}+(1-\alpha_{n})T_{i}(x_{1,n},x_{2,n}, \ldots ,x_{N,n}),\quad n=0,1,2, \ldots, $$
  *converges weakly to* $p_{i}$ *for all* $i=1,2,\ldots,N$, *where* $0< a\leq\alpha _{n}\leq b<1$ *for two constants* *a*, *b*.

### Proof

Conclusion (1) is obtained from Theorem 3.2. Next, we prove conclusion (2). From Lemma 4.4 we know that $(X^{N}, \|\cdot\|_{*})$ is a uniformly convex Banach space that satisfies Opial’s condition. It is easy to see that $C^{N}$ is a nonempty closed convex bounded subset in Banach space $(X^{N}, \|\cdot\|_{*})$. The nonexpansive mapping $T^{*}: C^{N}\rightarrow C^{N}$ is defined as in Theorem 4.2. By Theorem 1.2, for any given $x_{0} \in C^{N}$, the iterative sequence defined by

4.2 $$ x_{n+1}=\alpha_{n} x_{n}+(1-\alpha_{n})T^{*}x_{n} $$

converges weakly to a fixed point $p=(p_{1},p_{2},\ldots ,p_{N})$ of $T^{*}$, that is,

$$T^{*}(p_{1},p_{2},\ldots,p_{N})=(p_{1},p_{2}, \ldots,p_{N}). $$

Let

$$x_{n}=(x_{1,n},x_{2,n},\ldots,x_{N,n}), \quad n=0, 1,2,3, \ldots. $$

Then iterative scheme (4.2) can be rewritten as

$$u_{i, n+1}=\alpha_{n} u_{i,n}+(1-\alpha_{n})T_{i}(u_{1,n},u_{2,n}, \ldots,u_{N,n}),\quad n=0,1,2, \ldots, $$

for all $i=1,2, \ldots,N$.

By Lemma 4.4, for any $f_{1}, f_{2},\ldots,f_{N} \in(X, \|\cdot\|)^{*}$, we have that

$$F=(f_{1},f_{2},\ldots,f_{N}) \in \bigl(X^{N}, \|\cdot\|_{*}\bigr)^{*}. $$

Hence

$$F(x_{n})=\sum_{i=1}^{N}f_{i}(x_{i,n}), \quad n=0,1,2, 3,\ldots, $$

converges to

$$F(p)=F(p_{1},p_{2},\ldots,p_{N})=\sum _{i=1}^{N}f_{i}(p_{i}). $$

For any $1\leq i \leq N$, let $f_{j}=0$ for $j\neq i$. From the above result we have that $f_{i}(x_{i,n})\rightarrow f_{i}(p_{i})$ as $n\rightarrow \infty$. Hence $\{x_{i,n}\}$ converges weakly to $p_{i}$ for all $i=1,2,\ldots,N$. This completes the proof. □

### Theorem 4.6

*Let* *C* *be a nonempty closed convex subset of a real uniformly smooth Banach space* *X*, *and let* $T_{i}: C^{N}\rightarrow C$ *an* *N*-*varible nonexpansive mapping for all* $i=1,2,\ldots,N$. *Assume that* $(X^{N}, \|\cdot\|_{*})$ *is a uniformly smooth Banach space and the solution set of the system of operator equations* (4.1) *is nonempty*. *Let* $\{\alpha_{n}\} \subset[0,1]$ *be a sequence of numbers satisfying the following conditions*:

(C_1_): $\sum_{n=1}^{\infty}\alpha_{n}=\infty$;
(C_2_): $\lim_{n\rightarrow\infty}\alpha_{n}=0$;
(C_3_): $\lim_{n\rightarrow\infty}\frac{\alpha_{n+1}-\alpha_{n}}{\alpha_{n}}=0$.

*Then*, *for any given* $x_{0}=(x_{1,0},x_{2,0},x_{3,0},\ldots ,x_{N,0}) \in C^{N}$, *the iterative processes* $\{x_{n}\}$ *defined by*

$$x_{i, n+1}=\alpha_{n} u+(1-\alpha_{n})T_{i}(x_{1,n},x_{2,n}, \ldots ,x_{N,n}),\quad n=0,1,2, \ldots, u \in C, $$

*converges strongly to an element* $p_{i} \in C$ *for all* $i=1,2,\ldots,N$. *The element* $p=(p_{1},p_{2}, \ldots,p_{N})$ *is a solution of the system of operator equations* (4.1).

### Proof

It is easy to see that $C^{N}$ is a nonempty closed convex subset in the uniformly smooth Banach space $(X^{N}, \|\cdot\|_{*})$. The nonexpansive mapping $T^{*}: C^{N}\rightarrow C^{N}$ is defined as in Theorem 4.2. By Theorem 1.3, for any given $x_{0} \in C^{N}$, the iterative sequence defined by

4.3 $$ x_{n+1}=\alpha_{n} u+(1-\alpha_{n})T^{*}x_{n} $$

converges strongly to a fixed point $p=(p_{1},p_{2},\ldots ,p_{N})$ of $T^{*}$, that is,

$$T^{*}(p_{1},p_{2},\ldots,p_{N})=(p_{1},p_{2}, \ldots,p_{N}). $$

Let

$$x_{n}=(x_{1,n},x_{2,n},\ldots,x_{N,n}), \quad n=0, 1,2,3, \ldots. $$

Then iterative scheme (4.3) can be rewritten as

$$u_{i, n+1}=\alpha_{n} u_{i,n}+(1-\alpha_{n})T_{i}(u_{1,n},u_{2,n}, \ldots,u_{N,n}),\quad n=0,1,2, \ldots, $$

for all $i=1,2, \ldots,N$. Hence $\{x_{i,n}\}$ converges strongly to $p_{i}$ for all $i=1,2,\ldots,N$. This completes the proof. □

The concept of a coupled fixed point was introduced by Chang and Ma [20] in 1991. Since then, the concept has been of interest to many researchers in metrical fixed point theory [21–24]. In 2006, Bhaskar and Lakshmikantham [24] introduced the concept of a coupled fixed point in the setting of single-valued mappings and established some coupled fixed point results and found its application to the existence and uniqueness of solutions for periodic boundary value problems. In 2011, Berinde and Borcut [25] introduced the concept of a tripled fixed point for nonlinear mappings in complete metric spaces.

### Definition 4.7

([20])

Let *X* be a nonempty set. An element $(x_{1}, x_{2}) \in X \times X$ is called a coupled fixed point of mapping $T : X \times X \rightarrow X$ if

$$\textstyle\begin{cases} T(x_{1},x_{2})=x_{1}, \\ T(x_{2},x_{1})=x_{2}. \end{cases} $$

### Definition 4.8

([25])

Let *X* be a nonempty set. An element $(x_{1}, x_{2},x_{3}) \in X \times X \times X$ is called a tripled fixed point of a mapping $T : X \times X \times X \rightarrow X$ if

$$\textstyle\begin{cases} T(x_{1},x_{2},x_{3})=x_{1}, \\ T(x_{2},x_{3},x_{1})=x_{2}, \\ T(x_{3},x_{1},x_{2})=x_{3}. \end{cases} $$

### Definition 4.9

([7])

Let $(X,d)$ be a metric space, and let $T: X^{N}\rightarrow X$ be an *N*-variable mapping. An element $p\in X$ is called a multivariate fixed point if

$$p=T(p,p,\ldots,p). $$

Form the above results we get the following corollaries.

### Corollary 4.10

*Let* *X* *be a real uniformly convex Banach space*, *and let* *C* *a nonempty closed convex bounded subset of* *X*. *Let* $T: C^{2}\rightarrow C$ *be a two*-*variable nonexpansive mapping*. *Let*

$$\|x\|_{*}= \sqrt{ \|x_{1}\|^{2}+\|x_{2} \|^{2}} $$

*for all* $x=(x_{1},x_{2}) \in X^{2}$. *Assume that* $(X^{2}, \|\cdot\|_{*})$ *is a uniformly convex Banach space*. *Then* *T* *has a coupled fixed point*.

### Proof

Let $T_{1}, T_{2}: C^{2}\rightarrow C$ be defined by

$$T_{1}(x_{1},x_{2})=T(x_{1},x_{2}), \qquad T_{2}(x_{1},x_{2})=T(x_{2},x_{1}) $$

for all $(x_{1},x_{2}) \in C^{2}$. Then $T_{1}$, $T_{2}$ are two-variable nonexpansive mappings. By Theorem 4.2 there exists an element $(x_{1},x_{2}) \in C^{2}$ such that

$$\textstyle\begin{cases} T_{1}(x_{1},x_{2})=x_{1}, \\ T_{2}(x_{1},x_{2})=x_{2}, \end{cases} $$

that is,

$$\textstyle\begin{cases} T(x_{1},x_{2})=x_{1}, \\ T(x_{2},x_{1})=x_{2}. \end{cases} $$

Then $(x_{1},x_{2})$ is a coupled fixed point of *T*. This completes the proof. □

By using the same way as in Corollary 4.10, we can get Corollary 4.11.

### Corollary 4.11

*Let* *X* *be a real uniformly convex Banach space*, *and let* *C* *a nonempty closed convex bounded subset of* *X*. *Let* $T: C^{3}\rightarrow C$ *be a three*-*variable nonexpansive mapping*. *Let*

$$\|x\|_{*}= \sqrt{ \|x_{1}\|^{2}+\|x_{2} \|^{2}+\|x_{3}\|^{2}} $$

*for all* $x=(x_{1},x_{2}, x_{3}) \in X^{3}$. *Assume that* $(X^{3}, \|\cdot\| _{*})$ *is a uniformly convex Banach space*. *Then* *T* *has a tripled fixed point*.

### Corollary 4.12

*Let* *X* *be a real uniformly convex Banach space*, *and let* *C* *be a nonempty closed convex bounded subset of* *X*. *Let* $T: C^{N}\rightarrow C$ *be an* *N*-*variable nonexpansive mapping*. *Let*

$$\|x\|_{*}= \sqrt{ \sum_{i=1}^{N} \|x_{i}\|^{2}} $$

*for all* $x=(x_{1},x_{2}, \ldots,x_{N}) \in X^{N}$. *Assume that* $(X^{N}, \| \cdot\|_{*})$ *is a uniformly convex Banach space*. *Then* *T* *has a multivariate fixed point*.

### Proof

Let $T_{i}=T$ for $i=1,2,\ldots,N$. By Theorem 4.2 there exists an element $p=(p_{1},p_{2}, \ldots, p_{N})\in C^{N}$ such that

$$\textstyle\begin{cases} T(p_{1},p_{2},\ldots,p_{N})=p_{1}, \\ T(p_{1},p_{2},\ldots,p_{N})=p_{2}, \\ \ldots, \\ T(p_{1},p_{2},\ldots,p_{N})=p_{i}, \\ \ldots, \\ T(p_{1},p_{2},\ldots,p_{N})=p_{N}. \end{cases} $$

This implies that $p_{1}=p_{2}=\cdots=p_{N}$. Then *T* has a multivariate fixed point. This completes the proof. □

By Theorem 4.5 we get the following three corollaries.

### Corollary 4.13

*Let* *X* *be a real uniformly convex Banach space that satisfies Opial’s condition*, *and let* *C* *be a nonempty closed convex bounded subset of* *X*. *Let* $T: C^{2}\rightarrow C$ *be a two*-*variable nonexpansive mapping*. *Let*

$$\|x\|_{*}= \sqrt{ \|x_{1}\|^{2}+\|x_{2} \|^{2}} $$

*for all* $x=(x_{1},x_{2}) \in X^{2}$. *Assume that* $(X^{2}, \|\cdot\|_{*})$ *is a uniformly convex Banach space*. *Then*

1. *T* *has a coupled fixed point*;
2. *for any given* $x_{0}=(x_{1,0},x_{2,0}) \in C^{2}$, *the iterative sequences* $\{x_{1,n}\}, \{x_{2,n}\}\subset X$ *defined by*
  $$x_{1,n+1}=\alpha_{n} x_{1,n}+(1-\alpha_{n})T(x_{1,n},x_{2,n}) $$
  *and*
  $$x_{2,n+1}=\alpha_{n} x_{2,n}+(1-\alpha_{n})T(x_{2,n},x_{1,n}) $$
  *converge weakly to two elements* $p_{1}$ *and* $p_{2}$, *respectively*, *and* $(p_{1},p_{2})$ *is a coupled fixed point of* *T*, *where* $0< a\leq\alpha _{n}\leq b<1$ *for two constants* *a*, *b*.

### Corollary 4.14

*Let* *X* *be a real uniformly convex Banach space that satisfies Opial’s condition*, *and let* *C* *be a nonempty closed convex bounded subset of* *X*. *Let* $T: C^{3}\rightarrow C$ *be a three*-*variable nonexpansive mapping*. *Let*

$$\|x\|_{*}= \sqrt{ \|x_{1}\|^{2}+\|x_{2} \|^{2}+\|x_{3}\|^{2}} $$

*for all* $x=(x_{1},x_{2}, x_{3}) \in X^{3}$. *Assume that* $(X^{3}, \|\cdot\| _{*})$ *is a uniformly convex Banach space*. *Then*

1. *T* *has a tripled fixed point*;
2. *for any given* $x_{0}=(x_{1,0},x_{2,0},x_{3,0}) \in C^{3}$, *the iterative sequences* $\{x_{1,n}\}, \{x_{2,n}\}, \{x_{3,n}\}\subset X$ *defined by*
  $$\begin{aligned}& x_{1,n+1}=\alpha_{n} x_{1,n}+(1-\alpha_{n})T(x_{1,n},x_{2,n}, x_{3,n}), \\& x_{2,n+1}=\alpha_{n} x_{2,n}+(1-\alpha_{n})T(x_{2,n}, x_{3,n}, x_{1,n}), \\& x_{3,n+1}=\alpha_{n} x_{3,n}+(1-\alpha_{n})T(x_{3,n}, x_{2,n},x_{1,n}) \end{aligned}$$
  *converge weakly to three elements* $p_{1}$, $p_{2}$, $p_{3}$, *respectively*, *and* $(p_{1},p_{2},p_{3})$ *is a tripled fixed point of* *T*, *where* $0< a\leq\alpha _{n}\leq b<1$ *for two constants* *a*, *b*.

### Corollary 4.15

*Let* *X* *be a real uniformly convex Banach space that satisfies Opial’s condition*, *and let* *C* *be a nonempty closed convex bounded subset of* *X*. *Let* $T: C^{N}\rightarrow C$ *be an* *N*-*variable nonexpansive mapping*. *Let*

$$\|x\|_{*}= \sqrt{ \sum_{i=1}^{N} \|x_{i}\|^{2}} $$

*for all* $x=(x_{1},x_{2},\ldots,x_{N}) \in X^{N}$. *Assume that* $(X^{N}, \|\cdot\|_{*})$ *is a uniformly convex Banach space*. *Then*

1. *T* *has a multivariate fixed point*;
2. *for any given* $x_{0}=(x_{1,0},x_{2,0},x_{3,0},\ldots,x_{N,0}) \in C^{N}$, *the iterative sequence* $\{x_{n}\}\subset X$ *defined by*
  $$x_{n+1}=\alpha_{n} x_{n}+(1-\alpha_{n})T(x_{1,n},x_{2,n}, \ldots ,x_{N,n}),\quad n=0,1,2, \ldots, $$
  *converges weakly to a multivariate fixed point of* *T*, *where* $0< a\leq \alpha_{n}\leq b<1$ *for two constants* *a*, *b*.

By Theorem 4.6 we get the following three corollaries.

### Corollary 4.16

*Let* *C* *be a nonempty closed convex subset of a real uniformly smooth Banach space* *X*, *and let* $T: C^{2}\rightarrow C$ *be a two*-*variable nonexpansive mapping*. *Assume that* $(X^{N}, \|\cdot\|_{*})$ *is a uniformly smooth Banach space and the coupled fixed point set of* *T* *is nonempty*. *Let* $\{\alpha_{n}\} \subset[0,1]$ *be a sequence of numbers satisfying the following conditions*:

(C_1_): $\sum_{n=1}^{\infty}\alpha_{n}=\infty$;
(C_2_): $\lim_{n\rightarrow\infty}\alpha_{n}=0$;
(C_3_): $\lim_{n\rightarrow\infty}\frac{\alpha_{n+1}-\alpha_{n}}{\alpha_{n}}=0$.

*Then*, *for any given* $x_{0}=(x_{1,0},x_{2,0}) \in C^{2}$, *the iterative sequences* $\{x_{1,n}\}, \{x_{2,n}\}\subset X$ *defined by*

$$\begin{aligned}& x_{1,n+1}=\alpha_{n} u+(1-\alpha_{n})T(x_{1,n},x_{2,n}), \quad u \in C, \\& x_{2,n+1}=\alpha_{n} u+(1-\alpha_{n})T(x_{2,n},x_{1,n}), \quad u \in C, \end{aligned}$$

*converge strongly to two elements* $p_{1}$, $p_{2}$, *respectively*, *and* $(p_{1},p_{2})$ *is a coupled fixed point of* *T*.

### Corollary 4.17

*Let* *C* *be a nonempty closed convex subset of a real uniformly smooth Banach space* *X*, *and let* $T: C^{N}\rightarrow C$ *be an* *N*-*variable nonexpansive mapping*. *Assume that* $(X^{N}, \|\cdot\|_{*})$ *is a uniformly smooth Banach space and the tripled fixed point set of* *T* *is nonempty*. *Let* $\{\alpha_{n}\} \subset[0,1]$ *be a sequence of numbers satisfying the following conditions*:

(C_1_): $\sum_{n=1}^{\infty}\alpha_{n}=\infty$;
(C_2_): $\lim_{n\rightarrow\infty}\alpha_{n}=0$;
(C_3_): $\lim_{n\rightarrow\infty}\frac{\alpha_{n+1}-\alpha_{n}}{\alpha_{n}}=0$.

*Then*, *for any given* $x_{0}=(x_{1,0},x_{2,0},x_{3,0}) \in C^{3}$, *the iterative sequences* $\{x_{1,n}\}$, $\{x_{2,n}\}, \{x_{3,n}\}\subset X$ *defined by*

$$\begin{aligned}& x_{1,n+1}=\alpha_{n} u+(1-\alpha_{n})T(x_{1,n},x_{2,n}, x_{3,n}), \quad u \in C, \\& x_{2,n+1}=\alpha_{n} u+(1-\alpha_{n})T(x_{2,n}, x_{3,n}, x_{1,n}),\quad u \in C, \\& x_{3,n+1}=\alpha_{n} u+(1-\alpha_{n})T(x_{3,n}, x_{2,n},x_{1,n}), \quad u \in C, \end{aligned}$$

*converge strongly to two elements* $p_{1}$, $p_{2}$, $p_{3}$, *respectively*, *and* $(p_{1},p_{2},p_{3})$ *is a tripled fixed point of* *T*.

### Corollary 4.18

*Let* *C* *be a nonempty closed convex subset of a real uniformly smooth Banach space* *X*, *and let* $T: C^{3}\rightarrow C$ *be a three*-*variable nonexpansive mapping*. *Assume that* $(X^{N}, \|\cdot\|_{*})$ *is a uniformly smooth Banach space and the fixed point set of* *T* *is nonempty*. *Let* $\{\alpha_{n}\} \subset[0,1]$ *be a sequence of numbers satisfying the following conditions*:

(C_1_): $\sum_{n=1}^{\infty}\alpha_{n}=\infty$;
(C_2_): $\lim_{n\rightarrow\infty}\alpha_{n}=0$;
(C_3_): $\lim_{n\rightarrow\infty}\frac{\alpha_{n+1}-\alpha_{n}}{\alpha_{n}}=0$.

*Then*, *for any given* $x_{0}=(x_{1,0},x_{2,0},x_{3,0},\ldots ,x_{N,0}) \in C^{N}$, *the iterative processes* $\{x_{n}\}$ *defined by*

$$x_{ n+1}=\alpha_{n} u+(1-\alpha_{n})T(x_{1,n},x_{2,n}, \ldots ,x_{N,n}), \quad n=0,1,2, \ldots, u \in C, $$

*converges strongly to a multivariate fixed point of* *T*.

### Applied example 4.19

We consider the system of equations of trigonometric functions

4.4 $$ \textstyle\begin{cases} \sin(x_{1}+x_{2}+x_{3})=x_{1}, \\ \cos(x_{1}+x_{2}+x_{3})=x_{2}, \\ \sin^{2}(x_{1}+x_{2}+x_{3})=2x_{3}. \end{cases} $$

Let

$$\textstyle\begin{cases} T_{1}(x_{1},x_{2},x_{3})=\sin(x_{1}+x_{2}+x_{3}), \\ T_{2}(x_{1},x_{2},x_{3})=\cos(x_{1}+x_{2}+x_{3}), \\ T_{3} (x_{1},x_{2},x_{3})=\frac{1}{2}\sin^{2}(x_{1}+x_{2}+x_{3}). \end{cases} $$

Then $T_{1}$, $T_{2}$, $T_{3}$ are three nonlinear mappings from $[-\pi, +\pi]^{3}$ into $[-\pi, +\pi]$. On the other hand, we have that

$$\begin{aligned}& \bigl\vert T_{1}(x_{1},x_{2},x_{3})-T_{1}(y_{1},y_{2},y_{3}) \bigr\vert \\& \quad = \bigl\vert \sin(x_{1}+x_{2}+x_{3})- \sin(y_{1}+y_{2}+y_{3}) \bigr\vert \\& \quad \leq \bigl\vert (x_{1}+x_{2}+x_{3})-(y_{1}+y_{2}+y_{3}) \bigr\vert \\& \quad \leq \vert x_{1}-y_{1} \vert + \vert x_{2}-y_{2} \vert + \vert x_{3}-y_{3} \vert \\& \quad \leq\sqrt{ \frac{1}{3}\bigl( \vert x_{1}-y_{1} \vert ^{2}+ \vert x_{2}-y_{2} \vert ^{2}+ \vert x_{3}-y_{3} \vert ^{2} \bigr)}, \\& \bigl\vert T_{2}(x_{1},x_{2},x_{3})-T_{2}(y_{1},y_{2},y_{3}) \bigr\vert \\& \quad = \bigl\vert \cos(x_{1}+x_{2}+x_{3})- \cos(y_{1}+y_{2}+y_{3}) \bigr\vert \\& \quad \leq \bigl\vert (x_{1}+x_{2}+x_{3})-(y_{1}+y_{2}+y_{3}) \bigr\vert \\& \quad \leq \vert x_{1}-y_{1} \vert + \vert x_{2}-y_{2} \vert + \vert x_{3}-y_{3} \vert \\& \quad \leq\sqrt{ \frac{1}{3}\bigl( \vert x_{1}-y_{1} \vert ^{2}+ \vert x_{2}-y_{2} \vert ^{2}+ \vert x_{3}-y_{3} \vert ^{2} \bigr)}, \\& \bigl\vert T_{3}(x_{1},x_{2},x_{3})-T_{3}(y_{1},y_{2},y_{3}) \bigr\vert \\& \quad =\frac{1}{2} \bigl\vert \sin^{2}(x_{1}+x_{2}+x_{3})- \sin^{2}(y_{1}+y_{2}+y_{3}) \bigr\vert \\& \quad \leq\frac{1}{2} \bigl\vert \sin(x_{1}+x_{2}+x_{3})+ \sin(y_{1}+y_{2}+y_{3}) \bigr\vert \bigl\vert \sin (x_{1}+x_{2}+x_{3})-\sin(y_{1}+y_{2}+y_{3}) \bigr\vert \\& \quad = \bigl\vert \sin(x_{1}+x_{2}+x_{3})- \sin(y_{1}+y_{2}+y_{3}) \bigr\vert \\& \quad \leq \bigl\vert (x_{1}+x_{2}+x_{3})-(y_{1}+y_{2}+y_{3}) \bigr\vert \\& \quad \leq \vert x_{1}-y_{1} \vert + \vert x_{2}-y_{2} \vert + \vert x_{3}-y_{3} \vert \\& \quad \leq\sqrt{ \frac{1}{3}\bigl( \vert x_{1}-y_{1} \vert ^{2}+ \vert x_{2}-y_{2} \vert ^{2}+ \vert x_{3}-y_{3} \vert ^{2} \bigr)} \end{aligned}$$

for all $(x_{1},x_{2},x_{3}), (y_{1},y_{2}, y_{3}) \in[-\pi, +\pi]^{3}$. Hence $T_{1}$, $T_{2}$, $T_{3}$ are three-variable nonexpansive mappings from $[-\pi, +\pi]^{3}$ into $[-\pi, +\pi]$ in the uniformly convex Banach space $R=(-\infty ,+\infty)$. Since $R^{3}$ with norm

$$\bigl\Vert (x_{1},x_{2},x_{3}) \bigr\Vert = \sqrt{ |x_{1}|^{2}+|x_{2}|^{2}+|x_{3}|^{2}} $$

is a uniformly convex Banach space, by Theorem 4.5 the system of operator equations

$$\textstyle\begin{cases} T_{1}(x_{1},x_{2},x_{3})=x_{1}, \\ T_{2}(x_{1},x_{2},x_{3})=x_{2}, \\ T_{3}(x_{1},x_{2},x_{3})=x_{3} \end{cases} $$

has a solution $(p_{1}, p_{2}, p_{3})$, and the iterative sequences $\{x_{1,n}\}, \{x_{2,n}\}, \{x_{3,n}\}\subset X$ defined by

$$\begin{aligned}& x_{1,n+1}=\alpha_{n} x_{1,n}+(1-\alpha_{n})T_{1}(x_{1,n},x_{2,n}, x_{3,n}), \\& x_{2,n+1}=\alpha_{n} x_{2,n}+(1-\alpha_{n})T_{2}(x_{1,n}, x_{2,n}, x_{3,n}), \\& x_{3,n+1}=\alpha_{n} x_{3,n}+(1-\alpha_{n})T_{3}(x_{1,n}, x_{2,n},x_{3,n}) \end{aligned}$$

converge to elements $p_{1}$, $p_{2}$, $p_{3}$, respectively, where $0< a\leq \alpha_{n}\leq b<1$ for two constants *a*, *b*. Then the system of equations of trigonometric functions (4.4) has a solution $(p_{1}, p_{2}, p_{3})$, and the iterative sequences $\{x_{1,n}\}, \{x_{2,n}\}, \{x_{3,n}\}\subset X$ defined by

$$\begin{aligned}& x_{1,n+1}=\alpha_{n} x_{1,n}+(1-\alpha_{n}) \sin(x_{1,n}+x_{2,n}+x_{3,n}), \\& x_{2,n+1}=\alpha_{n} x_{2,n}+(1-\alpha_{n}) \cos(x_{1,n}+x_{2,n}+x_{3,n}), \\& x_{3,n+1}=\alpha_{n} x_{3,n}+(1-\alpha_{n}) \frac{1}{2}\sin ^{2}(x_{1,n}+x_{2,n}+x_{3,n}) \end{aligned}$$

converge to elements $p_{1}$, $p_{2}$, $p_{3}$, respectively.

### Discussion 4.20

An important contribution of this paper is to prove some existence theorems for solutions of certain systems of the multivariate nonexpansive operator equations and to calculate the solutions by using the generalized Mann and Halpern iterative algorithms in uniformly convex and uniformly smooth Banach spaces. To get the desired results, we first need to study the convexity and smoothness of Cartesian products of uniformly convex Banach spaces and uniformly smooth Banach spaces, respectively. On the other hand, we used a clever way to prove the main results. This method converts a non-self-mapping into a self-mapping such that the classical results can be used. Of course, in the main theorems, the assumptions that “$(X^{N}, \|\cdot\|_{*})$ is a uniformly convex Banach space” and “$(X^{N}, \|\cdot\|_{*})$ is a uniformly smooth Banach space” are still a limitation.

## Conclusions

### Conclusion 5.1

Let $(X, \|\cdot\|)$ be a real uniformly convex Banach space, and let *C* be a nonempty closed convex bounded subset of *X*. Let $T_{i}: C^{N}\rightarrow C$ be an *N*-variable nonexpansive mapping for all $i=1,2,\ldots,N$. Let

$$\|x\|_{*}= \sqrt{ \sum_{i=1}^{N} \|x_{i}\|^{2}} $$

for all $x=(x_{1},x_{2},\ldots,x_{N}) \in X^{N}$. Assume that $(X^{N}, \|\cdot\|_{*})$ is a uniformly convex Banach space. Then the system of operator equations

5.1 $$ \textstyle\begin{cases} T_{1}(x_{1},x_{2},\ldots,x_{N})=x_{1}, \\ T_{2}(x_{1},x_{2},\ldots,x_{N})=x_{2}, \\ \ldots, \\ T_{i}(x_{1},x_{2},\ldots,x_{N})=x_{i}, \\ \ldots, \\ T_{N}(x_{1},x_{2},\ldots,x_{N})=x_{N} \end{cases} $$

has a solution $p=(p_{1},p_{2}, \ldots, p_{N})\in C^{N}$.

### Conclusion 5.2

Let *X* be a real uniformly convex Banach space that satisfies Opial’s condition, and let *C* be a nonempty closed convex bounded subset of *X*. Let $T_{i}: C^{N}\rightarrow C$ be an *N*-variable nonexpansive mapping for all $i=1,2,\ldots,N$. Let

$$\|x\|_{*}= \sqrt{ \sum_{i=1}^{N} \|x_{i}\|^{2}} $$

for all $x=(x_{1},x_{2},\ldots,x_{N}) \in X^{N}$. Assume that $(X^{N}, \|\cdot\|_{*})$ is a uniformly convex Banach space. Then the system of operator equations (5.1) has a solution $p=(p_{1},p_{2}, \ldots, p_{N})\in C^{N}$, and for any $x_{0}=(x_{1,0},x_{2,0},x_{3,0},\ldots,x_{N,0}) \in C^{N}$, the iterative sequence $\{x_{i,n}\}\subset X$ defined by

$$x_{i, n+1}=\alpha_{n} x_{i,n}+(1-\alpha_{n})T_{i}(x_{1,n},x_{2,n}, \ldots,x_{N,n}),\quad n=0,1,2, \ldots, $$

converges weakly to $p_{i}$ for all $i=1,2,\ldots,N$, where $0< a\leq \alpha_{n}\leq b<1$ for two constants *a*, *b*.

### Conclusion 5.3

Let *C* be a nonempty closed convex subset of a real uniformly smooth Banach space *X*, and let $T_{i}: C^{N}\rightarrow C$ be an *N*-variable nonexpansive mapping for all $i=1,2,\ldots,N$. Assume that $(X^{N}, \|\cdot\|_{*})$ is a uniformly smooth Banach space and the solution set of the system of operator equations (5.1) is nonempty. Let $\{\alpha_{n}\} \subset[0,1]$ be a sequence of numbers satisfying the following conditions:

(C_1_): $\sum_{n=1}^{\infty}\alpha_{n}=\infty$;
(C_2_): $\lim_{n\rightarrow\infty}\alpha_{n}=0$;
(C_3_): $\lim_{n\rightarrow\infty}\frac{\alpha_{n+1}-\alpha_{n}}{\alpha_{n}}=0$.

Then, for any given $x_{0}=(x_{1,0},x_{2,0},x_{3,0},\ldots ,x_{N,0}) \in C^{N}$, the iterative processes $\{x_{n}\}$ defined by

$$x_{i, n+1}=\alpha_{n} u+(1-\alpha_{n})T_{i}(x_{1,n},x_{2,n}, \ldots ,x_{N,n}),\quad n=0,1,2, \ldots, u \in C, $$

converges strongly to an element $p_{i} \in C$ for all $i=1,2,\ldots,N$. The element $p=(p_{1},p_{2}, \ldots, p_{N})$ is a solution of the system of operator equations (5.1).
